# Congenital Pouch Colon with Double Meckel’s Diverticulae

**Published:** 2013-10-01

**Authors:** Praveen Mathur, Rahul Gupta, Anita Simlot, Ram Babu Goyal

**Affiliations:** Department of Paediatric Surgery, SMS Medical College, Jaipur, India; 1 Department of Obst. and Gynaecology, SMS Medical College, Jaipur, India

**Dear Sir**

A one-day-old boy weighing 2.4 kg was admitted with imperforate anus. On physical examination there was marked abdominal distension and palpable bladder. External genitalia were normal. A per urethral 6F feeding tube could be passed easily into the bladder. Invertogram suggested the presence of congenital pouch colon malformation. Exploration revealed type 1 complete colonic pouch and a bladder diverticulum. This diverticulum had a fistulous communication with distal end of pouch. Terminal ileum was directly opening into the pouch. Appendix was absent. Meckel’s diverticulum was found 40 centimeters proximal to terminal ileum. There was another diverticulum on anti-mesenteric border 10 cm proximal to Meckel’s diverticulum. No attempt was made to excise these asymptomatic and normal appearing diverticulae. Coloplasty of type I CPC was deferred due to poor general condition of the baby. Fistula was ligated and end pouch colostomy was done in left iliac fossa. Cystoscopic evaluation for obstructive uropathy could not be possible as child succumbed in the immediate post operative period. Autopsy was not performed.

**Figure:**
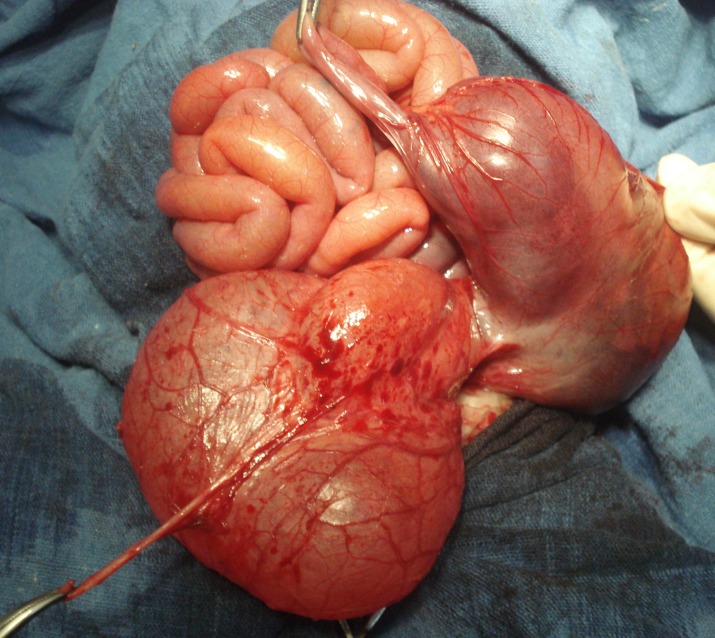
Figure 1: Operative photograph shows type I CPC, distended bladder, bladder diverticula and colo-diverticular fistula. A hemostat holds the terminal ileum. Note the absence of the appendix. Another hemostat holds the stay suture applied to bladder diverticulum

**Figure:**
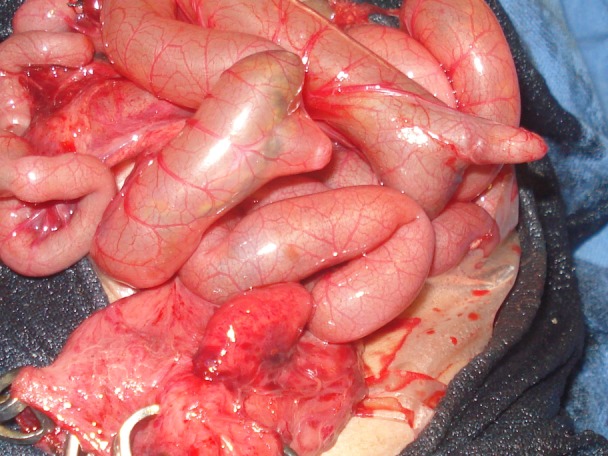
Figure 2: Operative photograph shows two Meckel’s diverticulae. Babcock forceps holds the decompressed pouch colon

**Figure:**
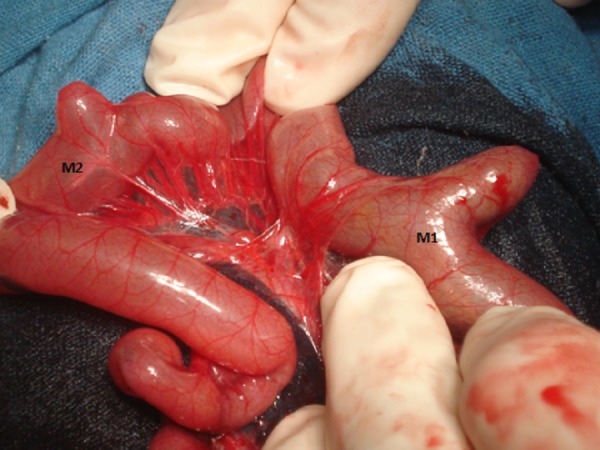
Figure 3: Operative photograph shows two Meckel’s diverticulae (M1 and M2) and the vasculature on mesenteric side

The congenital pouch colon is found to be associated with many genitourinary and gastrointestinal anomalies. A total 11 cases of Meckel’s diverticulum with CPC have been reported in literature. [1] Unusual association of pouch colon with another pouch, pouch colon to rectal atresia and pouch colon with duplicate bladder exstrophy has also been reported. [2, 3]

Coexistence of CPC and double Meckel’s diverticulum has not been reported earlier. We presume that this unusual presentation having two small intestinal diverticulae on anti-mesenteric border is a case of double Meckel’s diverticulum. The possible embryogenesis we suggest is that in early week of gestation, duplication of vitello-intestinal duct occurred and due to incomplete obliteration of both of these, double Meckel’s resulted.

## Footnotes

**Source of Support:** Nil

**Conflict of Interest:** None

